# Supplementary material for: A New Model Organism to Investigate Extraocular Photoreception: Opsin and Retinal Gene Expression in the Sea Urchin Paracentrotus lividus
Source: Cells. 2022 Aug 24;11(17):2636. doi: 10.3390/cells11172636 (PMC9454927; doi:10.3390/cells11172636)
Supplement: Supplementary file 1 [file cells-11-02636-s001.zip › cells-1870826 -Supplementary file S2.pdf]

Supplementary file S2: Investigated retinal genes in the sea urchin *P. lividus*

Gene ID	*P. lividus* gene name	*S. purpuratus* gene ID (https://www.echinobase.org/entry/)	Expression in *P. lividus* mature rudiment	Expression in *P. lividus* juvenile	Role in PRC development/ function
**PL39467**	*Ac/sc* (*Senseless*)	LOC593387	yes	NA	Pupal retinal development in *Drosophila* [78]
**PL31322**	*Atbf1*(*ZFHX3*)	LOC110979988	yes	NA	Modulates retinal sensitivity and circadian responses to light [74]
**PL36633**	*Atonal* (*Math5*)	LOC582139	yes	NA	retinal ganglion cell and optic nerve formation [76]
**PL25707**	*Barh*	LOC115919053	yes	NA	Differentially Regulates the Development of Retinal Amacrine and Ganglion Neurons [65]
**PL12878**	*Brn3 (Pou4f)*	LOC58164	yes	NA	Promotes retinal ganglion cell differentiation in chicken [68]
**PL12521**	*Dach*	LOC115918252	yes	NA	Key regulator of adult eye development in *Drosophila* [82]
**PL03830**	*Glis*	LOC593964	yes	NA	Controls precursor cell proliferation and differentiation in the developing mouse retina [75]
**PL11489**	*Hlf*	LOC110985920	yes	NA	Expressed in zebrafish adult photoreceptors[63]
**PL40150**	*IrxA*	NM_001129813	yes	NA	Controls retinal cell fate [72]
**PL00358**	*Islet*	LOC576365	yes	NA	Involved in cell specification and differentiation in the vertebrate retina [73]
**PL33304**	*NeuroD*	LOC756286	yes	yes	Controls differentiation of vertebrate photoreceptors [71]
**PL20621**	*Notch2*	LOC105437140	yes	NA	Regulates cell fate of retinal progenitors in vertebrates [61]
**PL09783**	*Opsin1* (ciliary opsin)	LOC110982350	no	yes	Vertebrate visual opsin in rods and cones[19]
**PL22079**	*Opsin2*	LOC100888012	yes	yes	Regulates backward swimming behavior in sea urchin larvae [77]

**PL31732**	*Opsin3.1* (Go-opsin)	LOC100891307	no	yes	Go-opsin mediates e.g. transduction cascade in scallop visual photoreceptors [20]
**PL31706**	*Opsin3.2* (Go-opsin)	LOC578047	no	yes	Another Go-opsin mediates the shadow reflex in the annelid *Plathynereis dumerilii* [21]
**PL22583**	*Opsin4* (rhabdomeric opsin)	LOC110980438	yes	yes	Visual opsin of many invertebrates eyes [19]
**PL21965**	*Opsin5*	LOC110980426	no	yes	Echinopsin with unknown function [18]
**PL10163**	*Otx*	LOC110974187	yes	NA	Required for tissue specification in the developing vertebrate eye [66]
**PL37631**	*Pax6*	LOC100892714	yes	yes	Required for maintaining multipotent state of vertebrate retinal progenitor cells [70]
**PL08229**	*Pp2ab56e*	LOC580795	yes	NA	Required for eye induction and eye field separation in vertebrates [64]
**PL00894**	*Rx*	LOC576952	no	NA	Regulates photoreceptor gene expression [67]
**PL08926**	*Six1/2*	LOC110974175	yes	NA	Part of the retinal determination gene network [69]
**PL08206**	*Six3*	LOC576281	yes	yes	Part of the retinal determination gene network [69]
**PL13313**	*Tbx3*	LOC592389	yes	NA	Required for vertebrate retina formation [62]

Citations are referring to main manuscript bibliography.
